# Risk of pancreatic cancer in patients undergoing surgery for chronic pancreatitis

**DOI:** 10.1186/s12893-019-0537-1

**Published:** 2019-07-08

**Authors:** Zhenjiang Zheng, Yonghua Chen, Chunlu Tan, Nengwen Ke, Binqing Du, Xubao Liu

**Affiliations:** Department of Pancreatic Surgery, West China Hospital, Sichuan University, GuoXue Lane No.37, Chengdu, 610041 Sichuan Province China

**Keywords:** Chronic pancreatitis, Pancreatic cancer, Surgery, Risk factor

## Abstract

**Background:**

Chronic pancreatitis (CP) is considered to be a risk factor for pancreatic cancer. A retrospective study was conducted to evaluate the incidence of pancreatic cancer after surgery for CP and to determine the risk factors.

**Methods:**

The patients who underwent surgery for histologically documented CP between January 2009 and December 2017 were reviewed. The baseline characteristics, operative data, postoperative complications, and follow-up information were analysed. We calculated standardized incidence ratio on the base of the incidence of pancreatic cancer in the standard population in China. The risk factor for pancreatic cancer was assessed using Cox regression.

**Results:**

Among 650 patients, pancreatic cancer was detected in 12 patients (1.8%) after a median follow-up of 4.4 years. The standardized incidence ratio of pancreatic cancer was 68.12 (95% CI, 35.20–118.99). Two independent risk factors for the development of pancreatic cancer in patients with chronic pancreatitis after surgery were identified: time interval to surgery [HR 1.005, 95% CI (1.002–1.008), *P* = 0.002] and de novo endocrine insufficiency [HR 10.672, 95% CI (2.567–44.372), *P* = 0.001].

**Conclusions:**

Patients who require surgery for CP are at a very high risk of developing pancreatic cancer. Early surgical intervention plays a protective role in the development of pancreatic cancer from CP. A high index of suspicion for pancreatic cancer should be maintained in CP patients with de novo postoperative diabetes after surgery.

## Background

Chronic pancreatitis (CP) is treated as one of risk factors affecting pancreatic cancer progression. The standardized incidence ratio (SIR) of pancreatic cancer in patients with CP was 11.8–20.22 with follow-up period after CP diagnosis of no less than two years [1–3]. As is well-known, it is usually difficult to make a differential diagnosis between pancreatic cancer and CP because these two kinds of diseases show similar radiological, biochemical as well as clinical features. [4]. On that account, there is a large possibility for pancreatic cancer being diagnosed as CP. For eliminating patients who were diagnosed as CP but were likely to present pancreatic cancer, a large number of studies which evaluated the relation of CP to pancreatic cancer excluded those who saw the development of pancreatic cancer within 2–5 years after being diagnosed as CP [5]. It is undoubted that the exclusion strategy had the function of minimizing the risk of misclassification while it was arbitrary and subjective to determine the time nodes. To minimize misdiagnosis bias, this study focused on patients who underwent surgery for histopathological confirmed CP. In addition, surgical treatment of CP can provide good pain control and achieve a satisfactory quality of life in the majority of patients in long-term follow up [6]. However, there are limited data available regarding pancreatic cancer after surgery for CP. We initiated a single-center cohort study to evaluate the incidence of pancreatic cancer after surgery for CP and to determine the risk factors.

## Methods

### Patients

Six hundred ninety two consecutive patients undergoing pancreatic operation between January 2009 and December 2017 for histologically confirmed CP were entered into a prospective database and retrospectively analysed. Two patients died of postoperative complications, 5 patients with autoimmune pancreatitis (AIP) and 1 patient underwent total pancreatectomy were excluded. 34 (4.9%) patients were lost to follow-up. Clinical data of the remaining 650 patients included following parameters: demographics; clinical presentation; operative data; morbidity and follow-up information. Before operation, all patients underwent magnetic resonance cholangiopancreatography (MRCP), endoscopic retrograde cholangiopancreatography (ERCP), computed tomography, tumor markers or other examinations. Histological confirmation helped to establish the diagnosis of pancreatic cancer. The study has obtained the approval from the Ethics Committee of West China Hospital. The permission to access the data was obtained from the institutional review board of our hospital (fetch code: apqe5932!).

### Definitions

Pancreatic fistula was defined as any measurable volume of drain fluid on or after postoperative day 3 with an amylase activity greater than 3 times the upper limit of normal serum amylase [7]. Biliary fistula was defined as bile drainage persisted for more than 5 days [8]. Exocrine insufficiency was defined as the presence of steatorrhea and/or the need for pancreatic enzyme replacement therapy. Endocrine pancreatic dysfunction was defined as the presence of diabetes. Time interval to surgery was defined as the time between initial diagnosis of CP and operative intervention.

### Surgical procedures

Operations were performed by specialists in pancreatic surgery. All procedures were performed in open surgical technique. There was no minimal-invasive operation in the study group. The procedures treating CP are composed of pancreaticoduodenectomy (PD), distal pancreatectomy (DP), duodenum-preserving pancreatic head resection (DPPHR), [9] as well as lateral pancreatojejunostomy (Partington procedure). The pancreatic cancer diagnosis was excluded by virtue of the examination of intraoperative frozen section of resection specimen during the initial operation time together with postoperative pathological paraffin sections diagnosis or the immunohistochemical examination.

For the treatment of pancreatic cancer, surgical resection including PD, DP and residual total pancreatectomy was performed in patients with localized and clearly resectable disease. Unresectable tumor was treated with palliative care.

### Follow-up

Patients or their relatives were followed up by outpatient visit or telephone contact every 6 months for the first 2 years and then yearly. Evaluations during the follow-up included questionnaires asking the pain status, the symptoms of diabetes or steatorrhea, and drug consumption. Furthermore, physical examinations, blood tests (e.g. CA19–9 levels) and abdominal CT scan were performed in the outpatient clinic of our hospital or a related hospital and clinic. The median follow-up was 4.4 years (ranging from 11 months to 10 years).

### Statistical analysis

Standard statistical programs (SPSS for Windows, version 17.0; Chicago, Ill) was used to carry out all statistical analyses. Continuous data were expressed in the form of means and standard deviations and Mann–Whitney *U* tests or Student’s *t*-test was used to compared these data. Fisher exact test together with Chi-square test were carried out to compared categorical data. The cumulative incidence as well as the overall survival rate of pancreatic cancer after surgery of CP were estimated via the Kaplan-Meier method. The log-rank test helped to test the difference. By definition, standardized incidence ratio (SIR) refers to the ratio of actual number of pancreatic cancers to estimated number in CP patient cohort, which could be used to indicate relative risk [1]. With regard to pancreatic cancer incidence in standard population of China, age stratification (based on an age interval of five years) on pancreatic cancer incidence in China in 2014 from the National Central Cancer Registry of China was used [10]. The univariate and multivariate Cox regression analyses were applied to assess the independent risk factors affecting the progression of pancreatic cancer after CP surgery [3]. All variables with univariable *P* < 0.1 were considered for the multivariable model. The resulting hazard ratios (HR) with its 95% confidence intervals (CI) were presented. Two sided *P* values were considered statistically significant at *P* < 0.05.

## Results

### Incidence and survival for pancreatic cancer development after surgery of CP

**Fig. 1:**
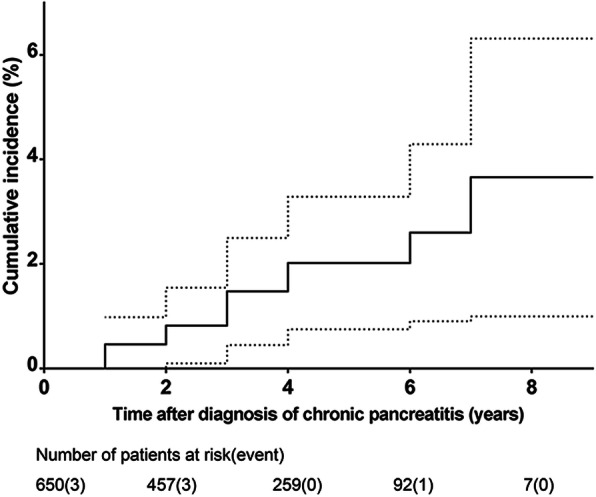
Cumulative incidence of pancreatic cancer after surgery of chronic pancreatitis. Dotted lines show 95% confidence interval

**Fig. 2:**
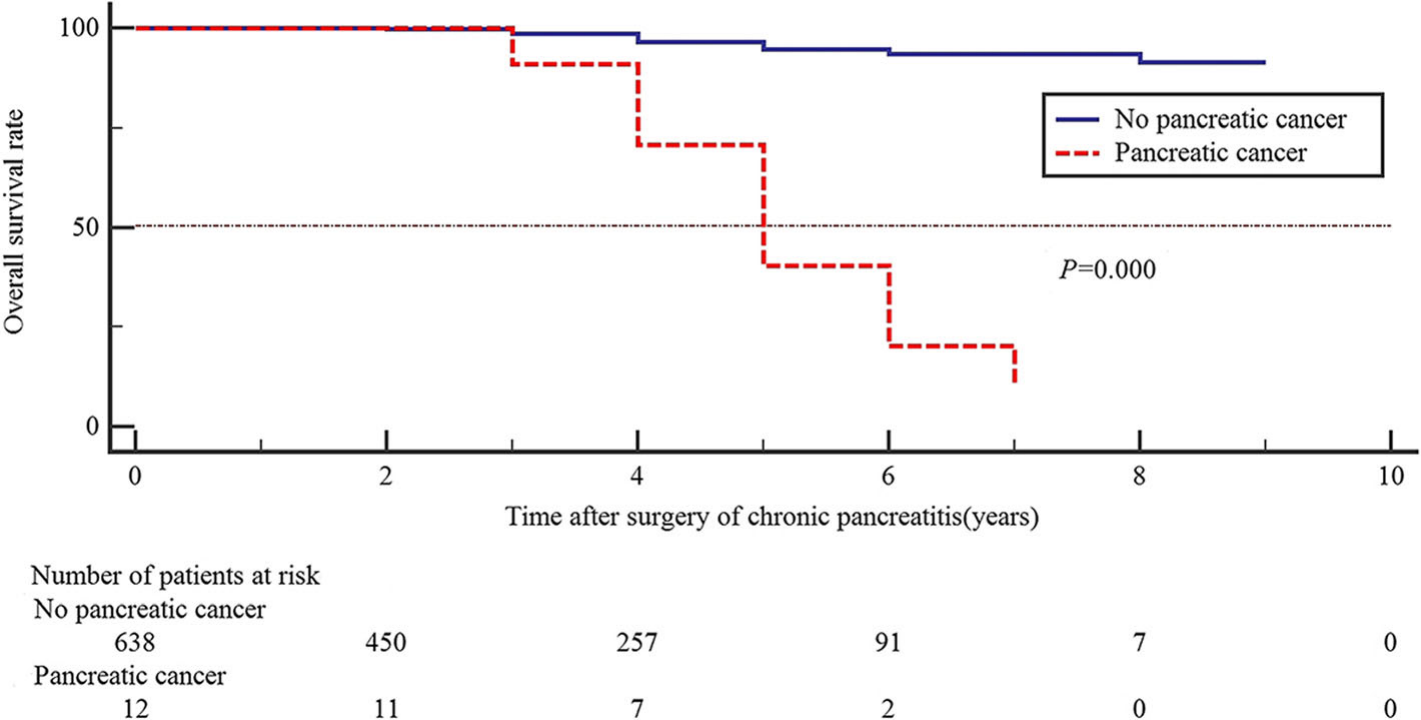
Comparison of overall survival rate between patients who had not developed to pancreatic cancer to those who had developed to pancreatic cancer

In the last follow-up time, 12 cases (1.8%) were diagnosed as pancreatic cancer based on the histology. The estimated number of pancreatic cancer patients reached 0.176, of which the SIR was 68.12 (95%CI, 35.20–118.99). Pancreatic cancer exhibited cumulative incidence of 1.48% (95% CI, 0.46–2.51%) at 3 years, 2.63% (95% CI, 0.93–4.32%) at 6 years and 3.71% (95% CI, 1.05–6.37%) at 9 years after CP surgery (Fig.1). Among the patients with pancreatic cancer, 8 patients underwent surgical resection involving PD in 6, DP in 1, and residual total pancreatectomy in 1. Four patients had unresectable pancreatic cancer at the time of diagnosis. Thirty one patients were deceased at the end of follow-up. Cause of death was related to pancreatic cancer in 9 patients (1.4%), other malignancies in 6 patients (1.1%), causes related to CP in 1 patient (0.2%), causes not related to CP in 7 (1.1%), and unknown causes in 8 (1.2%). Patients who had not developed to pancreatic cancer experienced better survival than those who had developed to pancreatic cancer [HR 17.071, 95% CI (7.816–37.285), *P* < 0.001]. The 1, 3 and 5 years survival rate were 100, 90.9 and 40.4%, respectively in patients who had developed to pancreatic cancer, as opposed to 100, 98.7 and 94.6% in patients who had not developed to pancreatic cancer (Fig.2).

### Baseline characteristics

According to development of cancer or not, the patients were divided into two groups, cancer group and non-cancer group. The baseline characteristics of the two groups were showed in Table 1. Patients in the cancer group had a longer time interval to surgery (*P* = 0.016). The other characteristics were comparable between the two groups.

**Table 1:** Baseline characteristics of the patients

Characteristics	Cancer group (%) (*n* = 12)	Non-cancer group (%) (*n* = 638)	*P*
Sex, (Male: Female)	9:3	501:137	0.728
Age, (yrs)	48.3 ± 7.2	45.4 ± 13.5	0.587
Time interval to surgery, (mons)	96.6 ± 164.4	36.4 ± 74.2	0.016
Etiology			0.751
Alcoholic	7 (58.3)	354 (55.5)	–
Idiopathic	5 (41.7)	222 (34.8)	–
Hereditary	0 (0)	62 (9.7)	–
Smoking			0.180
past	1 (8.3)	104 (16.3)	–
current	9 (75.0)	298 (46.7)	–
never	2 (16.7)	236 (37.0)	–
Alcohol consumption			1.0
past	1 (8.3)	55 (8.6)	–
current	9 (75.0)	441 (69.1)	–
never	2 (16.7)	142 (22.3)	–
Pain	11 (91.7%)	584 (91.5%)	0.987
Steatorrhea	2 (16.7%)	117 (18.3%)	0.882
Diabetes	4 (33.3%)	244 (38.2%)	0.729

### Operative data and postoperative complications

Operating time, blood loss, blood transfusion, type of surgery, complications in terms of pancreatic fistula (grade B and C), hemorrhage and bile fistula were showed in Table 2. Furthermore, complications according to the Clavien-Dindo classification (greater than grade ≥ 2) [11], hospital stay after operation is recorded. There was no difference in the operative and postoperative data between the cancer and non-cancer group.

**Table 2:** Operative data and postoperative complications

Characteristics	Cancer group (%) (n = 12)	Non-cancer group (%) (n = 638)	*P*
Operating time, (min)	200.8 ± 37.3	209.1 ± 47.3	0.643
Blood loss, (ml)	225.0 ± 121.5	219.1 ± 248.0	0.168
Blood transfusion	1(8.3%)	33(5.2%)	0.478
Type of surgery			0.190
PD	0 (0)	59 (9.2)	–
DPPHR	10 (83.3)	325 (50.9)	–
DP	1 (8.3)	66 (10.3)	–
Partington procedure	1 (8.3)	188 (29.5)	–
Pancreatic fistula	0 (0)	14 (2.2)	1.0
Hemorrhage	0 (0)	10 (1.6)	1.0
Biliary fistula	0 (0)	1 (0.2)	1.0
Complications (greater than grade ≥ 2)	1 (8.3)	11 (1.7)	0.202
Hospital stay after operation, (days)	7.9 ± 2.0	9.4 ± 6.9	0.571

PD, pancreaticoduodenectomy; DPPHR, duodenum-preserving pancreatic head resection; DP, distal pancreatectomy

### Pain, endocrine and exocrine functions at the last follow-up

Pain status, endocrine and exocrine functions at the last follow-up were showed in Table 3. In all, 460 (70.8%) patients reported no abdominal pain and 190 (29.2%) of the patients had abdominal pain including 4 de novo pain (0 [0%] of 1 patients in the cancer group vs. 4 [7.4%] of 54 patients in the non-cancer group, *P* = 1.0) and 12 worsening pain (1 [9.1%] of 11 patients in the cancer group vs. 11 [1.9%] of 584 patients in the non-cancer group, *P* = 0.202). A total of 274 patients (42.2%) had endocrine insufficiency at the last follow-up evaluation. The incidence of total postoperative endocrine insufficiency was not significantly different between the two groups. 26 of the 402 patients without preoperative diabetes had de novo endocrine insufficiency. The incidence of de novo endocrine insufficiency was higher in the cancer group, occurring in 4 (50.0%) of 8 patients in the cancer group and 22 (5.6%) of 394 patients in the non-cancer group (*P* = 0.001). In the last follow-up time, 35 cases exhibited exocrine insufficiency, thus 154 cases (23.7%) exhibited exocrine pancreatic insufficiency in total. The two groups showed no significant difference in term of the de novo or total postoperative exocrine insufficiency incidence.

**Table 3:** Pain, exocrine and endocrine functions at the last follow-up

Characteristics	Cancer group (n = 12)	Non-cancer group (n = 638)	*P*
Postoperative abdominal pain	6 (50%)	184(28.8%)	0.110
De novo pain	0 (0%) of 1 patient	4 (7.4%) of 54 patients	1.0
Worsening pain	1 (9.1%) of 11 patients	11 (1.9%) of 584 patients	0.202
Total postoperative endocrine insufficiency	8 (66.7%)	266 (41.7%)	0.083
De novo endocrine insufficiency	4 (50.0%) of 8 patients	22 (5.6%) of 394 patients	0.001
Total postoperative exocrine insufficiency	3 (25%)	151 (23.7%)	0.914
De novo exocrine insufficiency	1 (10.0%) of 10 patients	34 (6.5%) of 521 patients	0.497

### Risk factors affecting progression of pancreatic cancer following CP surgery

Twelve CP patients showed pancreatic cancer after surgery. The influences of baseline characteristics, perioperative data, postoperative pain, postoperative endocrine and exocrine functions, and abstinence from tobacco or alcohol after surgery were summarized in Table 4. Time interval to surgery, worsening pain, total postoperative endocrine insufficiency and de novo endocrine insufficiency were associated with high risk of pancreatic cancer in univariate analysis. Factors that affected pancreatic cancer development at the *P* < 0.10 level of significance by univariate analysis were included in a multivariate Cox regression model. After adjusting for these variables, time interval to surgery [HR 1.005, 95% CI (1.002–1.008), *P* = 0.002] and de novo endocrine insufficiency [HR 10.672, 95% CI (2.567–44.372), *P* = 0.001] were found to be independent variable influencing pancreatic cancer development.

**Table 4:** Results of univariable and multivariable regression for pancreatic cancer after surgery of CP

Variables	Univariate analysis		Multivariate analysis	
	HR (95%CI)	*P*	HR (95%CI)	*P*
Sex				
Male	0.811(0.219–2.997)	0.753		
Female	1.0			
Age, (yrs)				
≤ 50	1	0.386		
>50	0.561 (0.152–2.072)			
Time interval to surgery, (mons)	1.005 (1.002–1.008)	0.002	1.005(1.002–1.008)	0.002
Etiology		0.995		
Alcoholic	0.942(0.299–2.971)	0.919		
Idiopathic	1			
Hereditary	0.000	0.985		
Smoking		0.222		
past	1.215 (0.110–13.401)	0.874		
current	3.378 (0.730–15.642)	0.119		
never	1			
Alcohol consumption		0.719		
past	2.761(0.238–32.048)	0.417		
current	1.383(0.299–6.402)	0.678		
never	1			
Preoperative pain				
Yes	1.081 (0.139–8.381)	0.941		
No	1			
Preoperative steatorrhea				
Yes	1.044 (0.228–4.782)	0.958		
No	1			
Preoperative diabetes				
Yes	0.851 (0.255–2.840)	0.793		
No	1			
Operating time, min				
≤ 180	1	0.803		
> 180	1.165 (0.351–3.874)			
Blood loss, ml				
≤ 200	1	0.390		
> 200	1.694(0.509–5.641)			
Blood transfusion				
Yes	1.864(0.240–14.471)	0.552		
No	1			
Type of surgery				
PD	0.000	0.986		
DPPHR	6.671 (0.852–52.242)	0.71		
DP	2.857 (0.179–45.694)	0.458		
Partington procedure	1			
Pancreatic fistula				
Yes	0.48 (0.000–7,203,834.567)	0.752		
No	1			
Hemorrhage				
Yes	0.049(0.000–71,526,595.29)	0.779		
No	1			
Biliary fistula				
Yes	0.50 (0.000–2.309E+ 26)	0.926		
No	1			
Complications (greater than grade ≥ 2)				
Yes	5.338 (0.687–41.512)	0.109		
No	1			
Hospital stay after operation, (days)				
≤ 7	1			
> 7	0.803 (0.252–2.556)	0.710		
Postoperative abdominal pain				
Yes	2.457 (0.792–7.619)	0.120		
No	1			
De novo pain				
Yes	0.49 (0.000–6.085E+ 10)	0.832		
No	1			
Worsening pain				0.358
Yes	5.606 (0.721–43.560)	0.099		
No	1			
Total postoperative endocrine insufficiency				0.579
Yes	2.949 (0.883–9.851)	0.079		
No	1			
De novo endocrine insufficiency			10.672 (2.567–44.372)	0.001
Yes	13.106 (3.944–43.552)	0.000		
No	1			
Total postoperative exocrine insufficiency				
Yes	1.121 (0.303–4.144)	0.864		
No	1			
De novo exocrine insufficiency				
Yes	1.242 (0.159–9.681)	0.836		
No	1			
Give up smoking				
Yes	2.623 (0.790–8.714)	0.115		
No	1			
Give up drinking				
Yes	2.009 (0.637–6.340)	0.234		
No	1			

*HR* Hazard ratio, *CI* Confidence interval, *PD* pancreaticoduodenectomy, *DPPHR*, duodenum-preserving pancreatic head resection, *DP*, distal pancreatectomy

## Discussion

CP has been reported as one of risk factors affecting pancreatic cancer progression. Based on study by Löwenfels et al. [2], pancreatic cancer exhibited a SIR of 16.5 in a terrain of CP over 2 years of follow-up, and a SIR of 14.4 over 5 years of follow-up. As published by Hao et al. [3], pancreatic cancer in 1656 CP patients showed a SIR of 20.22 in China. According to a recent meta-analysis, 5 years after being diagnosed as chronic pancreatitis, the risk of pancreatic cancer increased by nearly eight fold [5]. In the study, we observed pancreatic cancer in 12 patients (1.8%) with histologically confirmed CP following surgery ina median follow-up of 4.4 years. The expected number of pancreatic cancer was 0.176, of which the SIR was 68.12. Pancreatic cancer exhibited a cumulative incidence of 3.71% (95% CI, 1.05–6.37%) at the end of follow-up. There are 2 risk factors regarding the pancreatic cancer,1) time interval to surgery; 2) de novo endocrine insufficiency.

CP is a kind of disease with extensive parenchymal fibrosis of pancreas and can be divided into obstructive form, calcifying form as well as steroid-responsive form [12]. As is well known, the inflammatory process may cause cancer, making people believe that pancreatic cancer can be caused by chronic inflammation of pancreatic parenchyma. The relationship shows similarity to that between esophageal cancer and Barrett’s epithelium, between hepatic cancer and cirrhosis, between cholangiocarcinoma and sclerosing cholangitis, and between colon cancer and ulcerative colitis [13]. Nevertheless, we are still unknown about the mechanism of the relationship between CP and pancreatic cancer. Based on reports, pancreatic acinar contains a sufficient activity level of Ras, which proves that CP is directly linked with pancreatic cancer [14]. In addition, both pancreatic cancer tissue and CP have same dysregulated signaling pathways, such as nuclear factor kappa B, cytokines, peroxisome proliferator-activated receptor-γ, as well as reactive oxygen species [15]. However, there is possibly no identical genetic susceptibility between pancreatic cancer and CP, at least in the variants with high frequency [16].

The surgical intervention indications of CP were composed of intractable pain, malignancy suspicion, as well as local complications [9]. CP patients who had not underwent surgery exhibited a much higher possibility in developing pancreatic cancer compared with those who received surgery [1]. This kind of diversity might be because the fibrotic pancreatic parenchyma was resected and pancreatic inflammation was relieved through surgical drainage. However, the SIR of pancreatic cancer in patients with CP after surgery found in our study was higher compared with other studies in which not all patients with CP underwent surgery [1–3]. This is may be because the age-specific incidence of pancreatic cancer is various in different countries and regions, result in the expected number of cancer cases was different. Thus, further studies evaluating the incidence of pancreatic cancer among patients with CP underwent surgery compared with no surgery are required. As reported by Lamme et al., early surgical drainage of chronic obstructive pancreatitis improved recovery of histology grades and pancreatic exocrine function compared to late surgical drainage in animal model [17]. A system review demonstrated that early surgery was benefit for postoperative pain relief, preservation pancreatic function and the need for further intervention in patient with CP [18]. Our study showed the time interval to surgery for CP is independent risk factor influencing pancreatic cancer development. Early surgical intervention plays a protective role in the development of pancreatic cancer from CP. In our study, the other independent risk factor of developing pancreatic cancer was de novo postoperative endocrine insufficiency. It was demonstrated that newly diagnosed diabetes was strong association with pancreatic cancer [19]. Therefore, it is possible that the presence of de novo postoperative endocrine insufficiency may be not only attribute to further ongoing parenchyma destruction by CP, but also as predictor of pancreatic cancer. It was analogous to considering periportal lymphadenopathy as a pioneer of various diseases [20].

In earlier studies [21, 22], alcohol consumption and smoking were considered as risk factors for the development of pancreatic cancer from CP. However, based on a pooled analysis on 12 cohort researches, there is no association between total alcohol intake and risk of pancreatic cancer [23]. According to study by Hao et al. [3],for people who smoke for over 60 packs each year, the risk of getting pancreatic cancer increased by 12 folds, while smoking without taking into account dose showed no significant difference. Kirkegård et al. [5] performed a meta-analysis and systematic review, finding the difficulty in assessing how alcohol and smoking affected CP developing into pancreatic cancer.. As demonstrated by Anderson et al. [21], the negative impact of alcohol and tobacco on pancreas will last for ten years. Our study demonstrated that abstinence from tobacco or alcohol after surgery of CP could not decrease risk factor for pancreatic cancer.

Despite of the application of advanced diagnostic approaches in recent years, it remains difficult to distinguish pancreatic cancer from CP. Based on existing study, nearly 5% of pancreatic cancers were misdiagnosed as CP in initial stage, the diagnosis of two-thirds of whom was delayed by two months to two years [24]. As we know, based on a majority of studies which evaluated the association of CP with pancreatic cancer, CP diagnosis was not determined according to the microscopic examination of the surgical specimens. Two studies assessed self-reported pancreatitis [25, 26], 4 studies evaluated patients via the International Classification of Diseases codes [27–30], and 1 study assessed patients from 1946 [2], in which period the risk of misdiagnosis was increased due to outdated diagnosis tools. Therefore, exclusion of patients who had been diagnosed within no less than two years after CP diagnosis would minimize the misdiagnosis bias [1]. The selection of lag period seems not to be evidence based and more likely arbitrary. Pathological examination is the gold standard for the diagnosis of diseases [31]. In our study, the diagnosis of CP was confirmed by histopathologic study of resection specimen, in order to eliminate misdiagnosis bias.

There are some limitations in our study. Firstly, this is a retrospective study that precision and completeness of data acquisition are difficult to control. Secondly, the data with regard to daily dose of smoking and alcohol consumption was lacking. Thus, whether smoking and alcohol consumption are dose-related to development of pancreatic cancer after surgery of CP has not been analysed. Thirdly, the limited size of pancreatic cancer patients may reduce statistical power.

## Conclusions

To sum up, according to the study, patients who require surgery for CP are more likely to develop pancreatic cancer. Time interval to surgery and de novo endocrine insufficiency were the risk factors affecting the pancreatic cancer progression in CP patients following surgery. The surgical intervention in early stage greatly affects CP developing into pancreatic cancer. It is necessary to maintain a high suspicion index for pancreatic cancer in CP patients showing de novo postoperative diabetes following the surgery.

## Data Availability

The datasets generated and/or analysed during the current study are available from the corresponding author on reasonable request.

## Abbreviations

CI: Confidence intervals
CP: Chronic pancreatitis
DP: Distal pancreatectomy
HR: Hazard ratios
PD: Pancreaticoduodenectomy
SIR: Standardized incidence ratio
